# Genetically modified plant–derived feed materials: results of official monitoring in Poland

**DOI:** 10.2478/jvetres-2025-0051

**Published:** 2025-09-24

**Authors:** Zbigniew Sieradzki, Małgorzata Mazur, Beata Król

**Affiliations:** Department of Microbiology of Food and Feed, National Veterinary Research Institute, 24-100 Puławy, Poland

**Keywords:** feed, GMO, maize, rapeseed, soybean

## Abstract

**Introduction:**

Highly efficient animal rearing would be impossible without the use of high-protein feed. In Europe the main source of feed protein has become soybean meal imported from South America, where the majority of it is genetically modified. The aim of the study was to determine the prevalence of genetically modified (GM) plant material in feed on the Polish market.

**Material and Methods:**

The study material consisted of feed materials and compound feed samples collected as part of the Official Feed Control Plan in 2018–2024. Methods recommended for use in official testing by the European Union Reference Laboratory for Genetically Modified Food and Feed were used to identify GM organism (GMO) presence.

**Results:**

Between 2018 and 2024, 171 (53.9%) positive samples were identified, all with GM soybean presence. No GM maize or GM rapeseed was identified. The majority of positive samples contained three GM soybean varieties: MON 40-3-2, MON 89788 and MON 87701. The results from samples taken at the eastern Polish border varied from one survey year to the next, revealing GMO presence ranging from 0% to 80%, and the entirety of the GMO content to be soybean.

**Conclusion:**

The high-protein soybean meal in poultry and pig farming is in part derived from GM soybeans grown in third countries. Other feed crops like maize and rapeseed are GMO-free or contaminated with GMOs only at low levels.

## Introduction

Poland has significant capacity in the production of poultry and eggs, which is used to meet the needs of its internal market and for export to the European Union and third countries. Maintaining this large poultry industry requires meeting the birds’ demand for high-protein feed. Modern and highly efficient livestock farming would be impossible without the use of feeds with optimal composition for the species and age of the animal and the purpose for which it is in production. Fattening animals for meat requires a high-protein, highly digestible feed with an optimal amino-acid profile and good conversion to animal body weight. Given the scale of animal production in Poland and the consequent need for well-balanced and uniform batches of finished compound feeds, animal feed manufacturers constantly need supplies of raw materials that are readily available, homogeneous and rich in the necessary ingredients required by the recipe. The source of feed protein in the EU and Poland is mainly plant raw material. Dependence on this source was caused by the withdrawal of meat and bone meal after the bovine spongiform encephalopathy (BSE) crisis and the ban on their use in livestock feed. The ban has recently been slightly modified and some concessions have been made, but the EU is not expected to make up the huge feed-protein deficit through these concessions. Poland, like other European countries, has limited possibilities for the production of plant-based fodder protein because of its climate. The available protein crops (rapeseed, lupine, fava beans and peas) have significant shortfalls in terms of protein quality and assimilability, there are no soybean varieties of which the yield would be economically satisfactory in the prevailing climate and the parties in the raw material market are not interested in developing an alternative to imports of raw materials. The emerging domestic soybean cultivation and soybean meal production is not able to meet the needs of the feed industry. Neither is commercially available rapeseed meal, which has characteristics unfavourable to its broad use in animal feed. The deficit in the availability of feed protein is the subject of research and implemented development programmes, both at the level of individual member states and the EU authorities. However, the developed and promoted domestic sources of feed protein are not able to replace imports of high-protein feed raw materials. Poland is not an exception in this aspect, because similar conditions apply to the EU as a whole, as well as to other countries. China, for example, is forced to make up the balance of its feed protein with imported soybean meal. European Union member states import approximately 40 million tonnes of soybean raw material each year, of which approximately 40% is unprocessed soybean and about 60% is soybean meal (10, 18, 23). The EU imports as much as 92% of its soy protein from third countries, including those using genetically modified organisms (GMOs) in their crops, with only 8% coming from domestic production. Poland imports around 1.4 million tonnes of feed protein each year, equivalent to 2.5 million tonnes in the form of imported soybean meal.

Cultivation of soybeans for feed purposes has developed in the USA and South American countries, where it is based on the use of genetically modified varieties (11). The asynchronous introduction of genetically modified plants into commercial cultivation worldwide results in a situation in which some GMOs authorised for use and cultivation in the USA or South American countries are not able to be used in the EU or Asian countries. A very low number of these genetically modified (GM) crops can legally reach the EU and be used, but their cultivation within Europe has been starkly limited in overall scale and is confined to a single crop in a few countries, particularly Spain, where farmers in several northern regions grow MON810, an insect resistant GM maize line (11, 17). This is because the use of GMOs for food and feed production is highly controversial in Europe, which is conservative in this respect. For this reason, restrictive rules have been introduced in the EU governing the placing of GMOs on the EU market and their use as food and feed ingredients, including the compulsory labelling of such products and official monitoring of the incidence of GMO inclusion (8, 9). The enforcement of EU and Polish regulations with regard to the use of GMOs in food and feed requires constant monitoring of the plant raw materials which may contain GM varieties. Plant species used in food and feed production which have GM varieties grown commercially somewhere are subject to GMO presence supervision. These species are mainly soybean, maize, canola, cotton, and to a lesser extent wheat, sugar beet and alfalfa. Products of GM plants that are authorised for use in the EU must carry labelling and consumer information about the presence of the GM ingredient. The European Commission maintains a register of GMO varieties authorised for use in food and feed or for cultivation and of GMOs withdrawn from the market or in the process of obtaining regulatory authorisation (7). Member states are obliged to keep watch for compliance with the legislation on the use and labelling of GMOs in food and feed. In Poland, the requirement for official control of feed for GMOs is carried out by the Veterinary Inspectorate through testing in Regional Veterinary Laboratories (RVLs) and National Reference Laboratories (NRLs) (13, 14). The Department of Microbiology of Food and Feed in the National Veterinary Research Institute (NVRI) is the Polish NRL for GMO feed containing rapeseed, cotton and microorganisms. The institute also screens feed in Poland officially for identification of GMOs including soybean and maize in feed. The purpose of this article is to report the prevalence of feed materials from GM plants determined in samples collected for official monitoring in Poland.

## Material and Methods

### Test samples

The study material consisted of feed materials and finished feed mixture samples collected as part of the Official Feed Control Plan in 2018–2024 by the Veterinary Inspectorate from the Mazowieckie, Lubelskie, Podkarpackie and Podlaskie voivodeships in central and eastern Poland. Sampling took place at farms and feed factories, from products in circulation on the market and from consignments imported to Poland from third countries (mainly at the border with Ukraine) on the basis of annual feed control plans prepared by the General Veterinary Inspectorate. In 2024, a significant proportion of the tests were of feed raw materials imported into Poland from Ukraine, hence the increase in 2024 samples from border controls. The total number of samples tested in 2018–2024 was 317, including 205 samples of soybeans and soybean meal, 26 of maize grain, 26 of rapeseed and rapeseed meal and 60 of finished compound feeds or complementary mixtures (Table 1).

**Table 1.** Number of analysed animal feed samples from 2018 to 2024

Year	Number of samples analysed per year				
	Soybean	Maize	Rapeseed	Compound feed	Total
2018	12	4	3	5	24
2019	27	3	2	7	39
2020	21	1	5	13	40
2021	29	2	5	8	44
2022	34	5	3	9	51
2023	37	8	5	8	58
2024	45	3	3	10	61
Total	205	26	26	60	317

### Detection and quantification by PCR and realtime PCR

Methods recommended for use in official testing by the European Union Reference Laboratory for Genetically Modified Food and Feed (EURL GMFF) were used for the detection and determination of GMO content. The methods used included protocols for GMO screening and others for the identification and quantification of individual GMO soybean, maize and rapeseed lines. Screening for GMOs and specific GM plant varieties was conducted by qualitative real-time PCR methods, which are detailed along with their target sequences in Table 2.

**Table 2.** Descriptions and types of targets for qualitative real-time PCR methods prescribed by the European Union Reference Laboratory for Genetically Modified Feed and Food (EURL GMFF) and used in official Polish monitoring of GM plant material in animal feed

Target type		Detection target	Target description	EURL GMFF unique identifier
Element		*nptII*	Neomycin phosphotransferase II	QL-ELE-00-002
		CaMV p35S	Cauliflower mosaic virus 35S promoter	QL-ELE-00-012
		t-NOS terminator	Cauliflower mosaic virus 35S promoter; nopaline synthase terminator	QL-ELE-00-013
		*bar*	phosphinothricin N-acetyl transferase from *Streptomyces hygroscopicus*	QL-ELE-00-014
		pFMV promoter	Figwort mosaic virus 35S promoter	QL-ELE-00-015
		*pat*	phosphinothricin N-acetyl transferase from *Streptomyces viridochromogenes*	QL-ELE-00-021
Construct		CTP2-CP4-EPSPS	Chloroplast transit peptide 2 and 5-enolpyruvylshikimate-3-phosphate synthase from *Agrobacterium tumefaciens* strain CP4	QL-CON-00-008
	Soybean	MON 87705	With glyphosate herbicide tolerance and improved fatty acid profile	QT-EVE-GM-003
		A2704-12	With phosphinothricin herbicide tolerance	QT-EVE-GM-004
		MON 40-3-2	With glyphosate herbicide tolerance	QT-EVE-GM-005
		MON 89788	With glyphosate herbicide tolerance	QT-EVE-GM-006
		MON 87701	With lepidopteran soybean pest resistance	QT-EVE-GM-010
		BPS-CV127-9	With imidazolinone herbicide tolerance	QT-EVE-GM-011
		Bt176	With lepidopteran soybean pest resistance, phosphinothricin herbicide tolerance and ampicillin resistance marker	QT-CON-00-007
	Maize	5307	With coleopteran maize pest resistance and mannose utilisation marker	QT-EVE-ZM-002
		NK603	With glyphosate herbicide tolerance	QT-EVE-ZM-008
		Bt11	With phosphinothricin herbicide tolerance and lepidopteran maize pest resistance	QT-EVE-ZM-015
		T25, MON 88017	T25: with phosphinothricin herbicide tolerance and ampicillin resistance marker MON 88017: with glyphosate herbicide tolerance and coleopteran maize pest resistance	QT-EVE-ZM-016
		MON 810	With lepidopteran maize pest resistance, glyphosate herbicide tolerance and neomycin/kanamycin resistance marker	QT-EVE-ZM-020
	Rapeseed	T45	With phosphinothricin herbicide tolerance	QT-EVE-BN-001
		Ms8	With phosphinothricin herbicide tolerance and male sterility trait	QT-EVE-BN-002
		Rf3	With phosphinothricin herbicide tolerance and fertility restoration trait	QT-EVE-BN-003
		Gt73	With glyphosate herbicide tolerance	QT-EVE-BN-004
		73496	With glyphosate herbicide tolerance	QT-EVE-BN-009
		MON 88302	With glyphosate herbicide tolerance	QT-EVE-BN-010

Once the samples potentially positive for GMOs were identified, the profiles of the screening elements were examined (using the algorithm from the EURL GMFF) to identify possible presences of specific GM events. Specific methods for GM plant varieties were subsequently applied allowing the detection and quantification of GMOs. The methods used for GMO identification and quantification are also listed in Table 2.

Detection and determination of GM soybeans were carried out on a 7500 real-time PCR system (Applied Biosystems, Middletown, CT, USA) in a 25 μL volume containing 1× TaqMan Universal Master Mix, a fixed volume of each primer, a fixed volume of TaqMan or minor groove binder probe and 5 μL of DNA. All primers and probes were synthesised and purified by high-performance liquid chromatography by Genomed (Warsaw, Poland).

## Results

European Union law obliges food and feed producers to inform the customer of the inclusion of GMO content above 0.9% of the total ingredients on the label or documentation of the goods. This legislative threshold is also appropriate for discrimination of positive and negative samples; thus, the samples described in the results as positive contained >0.9% GM soybean, maize or rapeseed by weight. Between 2018 and 2024, 171 (53.9%) positive samples were identified among all of the feed samples analysed (Table 3). No GMO lines not authorised for use in the EU were found in the positive samples. The most common positive samples were soybean and derived product materials such as soybean meal, soybean cake, *etc*., which were collectively included as soybean matrix in the test results. The percentage of samples positive for GM soybean matrix was 72.7% (149 positive samples out of 205 samples tested). In the ready-to-use complete feeds or supplementary mixes included in the results as compound feeds, positive samples accounted for 36.7% (22 out of 60). Compound feed samples were found positive solely because of GM soybean meal use in the production of the feed batch. Completely different test results were obtained for maize and rapeseed matrix: none of the samples tested made from maize (26 samples) or rapeseed (26 samples) had GMO content above 0.9%. Therefore, all such samples were classified as negative in the test results.

**Table 3.** Results of genetically modified organism analyses in all commercial Polish animal feed samples from 2018 to 2024

Matrix	Samples			Percentage of positive samples
	Total number	Positive	Negative	
Soybean	205	149	56	72.7
Maize	26	0	26	0.0
Rapeseed	26	0	26	0.0
Compound feed	60	22	38	36.7
Total	317	171	146	53.9

**Fig. 1.**
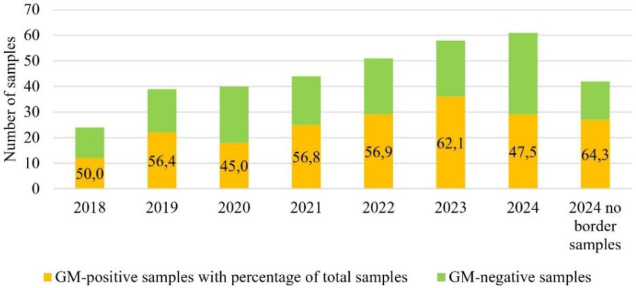
Numbers of positive and negative samples with positive percentages of the total pool of samples

The percentages of positive samples in each year of the study ranged from 45.0% in 2020 to 62.1% in 2023, and even up to 64.3% in 2024 when samples taken from Poland–Ukraine border controls were excluded (Fig. 1).

Among the negative samples, about 10% were cases where GMOs were found at levels below the limit of quantification (LOQ) of the methods used in the study. Depending on the method, the LOQ was from 0.04% to 0.07% GMO. An even smaller percentage were results where the presence of GMO was found between the method’s LOQ and the legislative level (*i.e*. in a range of 0.04/0.07% to 0.9%).

**Fig. 2.**
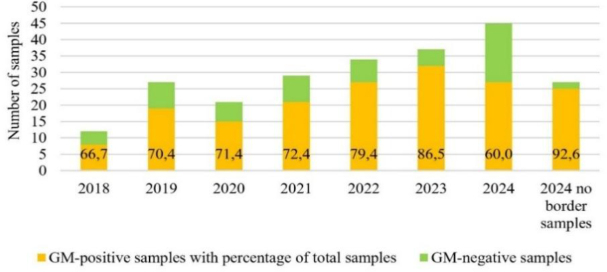
Numbers of positive and negative samples with positive percentages of all soybean matrix samples in 2018–2024

Considering the distribution of positive results by year of testing for each plant species or feed matrix analysed, it can be seen that there was vastly more GMO soybean among soybean feed samples than GMO maize among maize samples or GMO rapeseed among rapeseed samples. The proportion of GMO-positive soybean samples gradually increased from 66.7% in 2018 to 86.5% in 2023 (Fig. 2). Moreover, excluding samples from Poland’s eastern border with Ukraine in 2024, this percentage was as high as 92.6%. Approximately 98% of positive samples from 2018 to 2024 contained GM soybean of the MON 40-3-2, MON 89788 and/or MON 87701 events. The combination of these three GM soybean lines was found most frequently in the soybean matrix samples tested. The data for the individual years show that in 2018 the combination of MON 40-3-2 and MON 89788 lines was the most frequent positive result. In 2019, combinations involving four varieties (MON 40-3-2, MON 89788, MON 87701 and A2704-12) became the predominant result types. From 2020 to 2024, the three-variety combination of MON 40-3-2, MON 89788 and MON 87701 became the predominant result type.

**Fig. 3.**
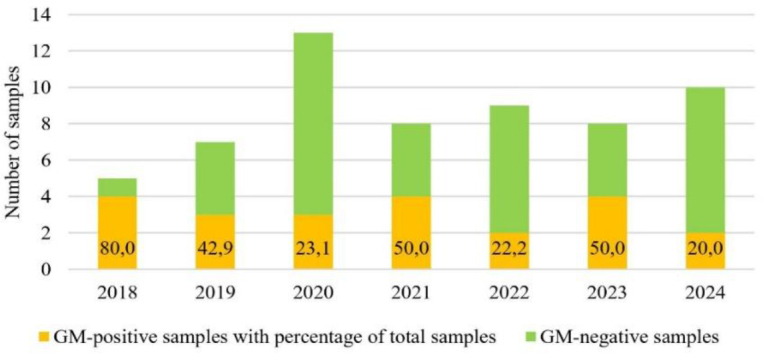
Number of positive and negative samples with positive percentages of all compound feed matrix samples in 2018 –2024

The results for the compound feed matrix showed that the percentage of positive samples varied, being highest in 2018 at 80.0% and ranging between 22.2% and 50.0% in subsequent years (Fig. 3). All positive samples of this matrix were so because of the presence of GM soybean. The detected profile of GM soybean lines corresponded to the profile in the soybean matrix and showed the same three lines – MON 40-3-2, MON 89788 and MON 87701 – to be the largest presences. No GMO maize or rapeseed content above 0.9% was found in any of the compound feeds.

**Fig. 4.**
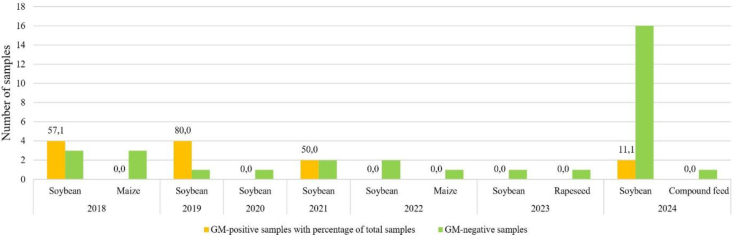
Numbers of positive and negative samples with positive percentages of all border inspection samples in 2018–2024

The results of samples taken from batches of feed raw materials or finished feed mixtures from controls at the eastern Polish border varied from one survey year to another (Fig. 4). The number of samples tested also varied and depended on the border control plans of the Veterinary Inspectorate at the time, or, starting in 2020, on the results of GMO screening tests on samples from the border in RVLs. In 2024, the number of border samples increased because the Veterinary Inspectorate was issued a special order to perform such tests by the NVRI. This order concerned samples of soybean meal imported from Ukraine into Poland. From 2018 to 2024, border screening of feed materials for GMOs was mostly undertaken on imports of soybean and its derivatives, and such samples were analysed annually. The percentage of positive soybean samples taken at border control points varied from 0% (in 2020, 2022 and 2023), through approximately 50% (in 2018 and 2021) to 80% (in 2019). There was a clear trend in the predominance of negative results from soybean samples in 2022 and subsequent years, with no positive samples found in 2022 or 2023 and only 11.1% of samples found positive in 2024. Maize samples were tested in 2018 and 2022, and none of these samples were positive for GM maize. Similar negative results were obtained for rapeseed samples from batches imported into Poland in 2023.

## Discussion

The results of the 2018–2024 survey indicate that 53.9 per cent of the feed samples contained GMOs and were appropriately labelled. An increase in the percentage of positive samples was observed each year of the study. This increase was of a few percent per year, from a level of 50% in 2018 to 62.1% (or even 64.3% without eastern Polish border-control results) in 2024. The observed increase in the use of GMOs over the six-year period was 12.1%, with a maximum of 14.3% after data correction in 2024. The results for the total feed samples are closely correlated with the results for one of the matrices analysed in the study – soybean and products made from it. For this particular matrix, the percentage of GM-positive samples showed an even more pronounced upward trend, from 66.7% in 2018 to 86.5% in 2023 and even 92.6% in 2024 (excluding border-control samples). Here, the aggregate increase was 19.8%, with a maximum of 25.9% after data correction in 2024. Considering the results from our previous soybean matrix surveys from 2004–2017 (24), it can be seen that after a periodic increase in negative results between 2017 and 2018 (30% and 33% of results negative, respectively), there was a renewed increase in the proportion of GM soybean used in Poland. Such a correlation of results between the use of GM soybean for soybean-derived feed and the percentage of feed-mixture samples positive for GMOs also indicates the high share of soybean meal in the production of mixtures in Poland. This is understandable given the type and extent of livestock farming in Poland.

Poland is one of the main European producers of poultry and eggs, and to a lesser extent of pork, which imposes a large demand for high-protein feed. The main source of feed protein in Poland and in the EU as a whole is soybean meal, due to uniform regulations and agricultural production conditions. Although Europe has never produced soybean on a large scale because of climatic conditions and the traditional raising of other crops, after the BSE crisis it began extensively importing soybean and soybean meal for feed purposes. The same path was obligatory for Poland to follow, because the country’s huge demand for feed protein to meet the growing needs of the feed industry necessitated the import of soybean meal. Domestic sources of feed protein in the form of legumes or rapeseed were not and are still not able to meet the demand for protein-rich raw materials. In addition, the protein from soybean has a diverse and balanced amino acid composition and is easily digestible by different animal species.

Genetically modified organism–free soybean has never been readily available for feed use in Poland. Nevertheless, in 2017–2018, many meat and milk processing plants in Poland used internal GMO-free production systems, hoping to increasing interest in non-GMO products and improve their brand image. The voluntariness and discretionary nature of such systems fared poorly in the Polish food market which is mainly oriented towards low product price, and the systems lost relevance under the law on GMO-free production introduced in Poland in 2020 (14). Low interest on the part of consumers in goods with a ‘GMO-free’ label, mainly for economic reasons, the aforementioned lack of readily available GMO-free soya, and the obligation upon farmers to make up feed according to the rules of the milk processing plants, contributed to the decline in interest among livestock farmers. This is reflected in the results of our research as well as in the limited supply of such products on the domestic market. Production which is certified ‘GMO-free’ in Poland outputs more expensive, niche products with a limited scope of application, chosen by customers oriented towards organic production. The small extent of production of this feed is also partly influenced by the conditions of the European market, where Polish poultry and eggs find many buyers. Polish exports of poultry and eggs have to be cost competitive and therefore rely on the most readily available and cheapest feed protein. This is protein derived from GM soybean. Furthermore, analytical laboratories still do not have the technical capacity to differentiate non-GMO feed used in rearing one animal from GMO feed used in rearing another when they only have the finished animal product to test. Other researchers stated clearly in 2018 that direct analysis of commercial animal-derived food products did not allow it to be established whether the original animal was fed any GMO-derived feed products (21). This has remained unchanged to the time of publication of the present investigation.

Poland’s reliance on imported soybean, of which a notable proportion consists of GM varieties, makes regulatory compliance especially significant. The number of non-compliant samples from the feed pool inspected for GMO declarations was very low, which meant that the customer was correctly informed, as required, about the GMO content in feed. The results of our study are comparable to those of other authors: Kleter *et al*. (18) stated that at the time of writing in 2018, an estimated over 90% of feed materials in EU were labelled as containing GMOs or GMO-derived materials. This high labelling rate corresponds to the widespread presence of major GM varieties in imported soybean. The presence of the Roundup Ready MON 4-03-2 soybean variety, the foundational GM soybean that dominates global production, is common in food and feed in many countries, even in some on the continent of Europe, according to reports of other authors (6, 15, 20, 22, 25). Deliberate cultivation of GM soybean was identified in Romania after its accession to the EU in 2007, which was likely linked to the pre-accession cultivation of MON 40-3-2 (26). This same variety was detected in 96 out of 111 soybean samples collected from six administrative regions in Ukraine. Ukrainian researchers concluded that GM crops were grown and sold there (12).

Our test results are comparable to those obtained in Germany. The Lower Saxony State Office for Consumer Protection and Food Safety (LAVES) stated on its official testing results website that in 2004, 27% of samples (31 out of 115 tested) contained MON 40-3-2 soybean above 0.9%, in 2005 15% did (10 out of 67), in 2006 28% (17 out of 60), in 2007 37% (11 out of 30) and in 2008 59% (13 out of 22) crossed the content threshold (19). Data for LAVES testing of GM maize over the same period shows that there were no samples containing more than 0.9% GMO. For rapeseed, only one GMO-positive sample yielding evidence of the Gt73 and LLP lines of Monsanto and Falcon rapeseed was found in 2006 and 2008, which in 2006 corresponded to 10% of the total pool of samples tested for this (1 in 10) and in 2008 to 7% (1 in 14). Summarising the results of its research at the time, LAVES concluded that although the number of feed samples tested was much smaller than the number of food samples tested for GM soybean ingredients, this result confirmed the trend that feed very often contained raw materials derived from GMOs. Similar results from the same country were found by the Bavarian State Office for Health and Food Safety (LGL). The testing website of the LGL indicates that between 2007 and 2014, GMO content of >0.9% was found in 37% of single-component feed samples, which broke down as 96 out of 132 soybean samples (73%), 5 out of 35 maize samples (14%) and 4 out of 31 rapeseed samples (13%). The website also provided parallel data for compound feed matrices: GMOs were found in 23 out of 82 complete mix samples (28%) and 34 out of 151 complementary mix samples (23%) (2). The data from 2015–2023 refer only to the number of non-compliant samples, *i.e*. without the GMO labelling which they should have had, and the number of such samples in Bavaria did not exceed a few percent per year. Another German authority with an official mandate – the Chemical and Veterinary Investigation Office of North Rhine-Westphalia – examined 64 feeds for genetically modified plants in 2017 (3). These were 56 samples with soybean, maize, rapeseed, wheat and linseed components, for which no genetic modification was declared, and two feeds that were labelled as containing genetically modified components from soybean. Of the unlabelled feeds, around 89% contained no GM material or only traces of it. One (1.8%) of the unlabelled feeds contained genetically modified components comprising more than the labelling threshold of 0.9%. This was a compound feed in which the approved soybean lines MON 40-3-2, A2704-12, A5547-127, MON87708, MON89788 and MN87701 were detected. Contents of approximately 30% were found for MON 40-3-2, 27% for MON89788, 9% for A2704-12 and 1.6% for MON87708. The contents determined for A5547-127 and MON87701 were below 0.9%. Soybean was not listed as an ingredient in this sample, but its content of 35%–40% was determined microscopically. No genetically modified plants not approved in the EU were detected in any of these feed samples, and no unauthorised transgenic lines were found in feed labelled with genetically modified ingredients. In 2006, the same authority found 17% of samples positive for GMOs in feed, in 2007 7%, in 2008 10.6%, in 2009 10.1%, in 2010 4.8%, in 2011 5%, in 2012 3%, in 2013 7.6%, in 2014 5.4%, in 2015 3.3% and in 2016 5.6%. From 2006 to 2013, the main GMO found in feed was soybean line MON 40-3-2, and from 2014 to 2017 they were lines MON 40-3-2, MON89788 and A2704. In 2012, a positive sample was also found for the GMO oilseed rape line Gt73.

Although our results from Poland are similar or comparable to those found in Germany, even for GM events in positive samples, EU member states’ or European countries feed markets may differ in GMO presence. Official data from Switzerland, operating under a different regulatory framework, indicated a low presence of GMOs in feed samples of domestic manufacture (4, 5, 16). In that country in 2022, no single-ingredient sample among 299 tested for the presence of GMOs in livestock feed was non-compliant (4). The same data for 2021 tests showed that there were no non-compliant samples in the total of 306 tested (5). In contrast, in 2020, GMO rapeseed was found in 2 feed samples out of 183 tested (16). For compound feed, in 2022, 2 samples out of 75 tested were found to contain GM rapeseed above the Swiss limit of 0.5%, while in 2020, GMOs were detected in 3 out of 114 (in bird feed). In another category – compulsorily GMO-free organic feed – no GMO contamination was detected in 2022 in 80 samples tested, in 2021 in 105 samples tested or in 2020 in 95 samples tested. Similarly, negative results were found for feed imported into Switzerland, where no sample containing GMOs was detected in 2022 in 62 samples tested or in 2021 in 57 samples tested. Similar test results of official feed testing with very low level of GM positive results were obtained by an EU member state authority – AGES in Austria – in 2018–2020. They indicated labelling problems with 2 samples each year out of 192 in 2018, 143 in 2019 and 177 in 2020 (1).

Results can vary because of the country, matrix, specific character of a market and political matters in which the crop market is implicated. In the 2024 data, in contrast to other years, we have cited the results without those of additional Poland–Ukraine border controls. The decision to conduct the analysis in this way gave due regard to their uniqueness and the lack of counterpart results from previous years. The results from the additional border controls clearly indicated that the only raw feed materials imported across the Polish–Ukrainian border in this period were those for which the risk of being challenged by the Polish side was as low as possible. Consequently, there was no presence in soybean of genetic modifications which could be inconsistent with the declared content and quality of the raw material. Furthermore, this was an unprecedented situation without equivalent previous years’ data, because prior to 2024 samples from the border were tested in much lower numbers and under routine rather than special control plans. Hence our interpretation of the results, which took into account the uniqueness of these tests and their significant impact on the assessment of the overall results. The validity of this concept is confirmed by the analytical results for the soybean matrix in 2024, where the inclusion of border samples reduced the proportion of GM soybean from 93% to 48% (a difference of 45%) for the total soybean samples analysed, and similarly reduced this proportion from 67% to 42% (a difference of 25%) for total feed. These are incomparably greater year-on-year changes than between any other pair of years in the study.

The significant proportion of feed raw materials derived from genetically modified plants in feed in Poland confirmed by our research has determined another postponement, this time until 1 January 2030, of the ban on the use of GMOs in animal nutrition in Poland (13). The amendment to the act on animal feedstuff assumes that the postponement of the ban will allow an assessment of how the situation in the protein crop market will change over the five-year period of 2025– 2030. In addition, the time is assumed adequate to allow feed companies to rebuild their existing production procurement and distribution systems and adapt their recipes and processing lines.

## Conclusion

Our results of testing feed for the presence of GMOs in Poland clearly indicated the source and origin of GMOs in feed. The use of high-protein soybean meal in poultry and pig farming is related to the use of GM soybean grown in third countries. Other feed crops like maize and oilseed rape are GMO-free or only a small percentage of samples is contaminated with GMOs at low levels. Despite the presence of GMOs in the feed market, the amount of non-compliant samples collected for official control was very low.
